# Epileptiform activity in mouse hippocampal slices induced by moderate changes in extracellular Mg^2+^, Ca^2+^, and K^+^

**DOI:** 10.1186/s12868-021-00650-3

**Published:** 2021-07-23

**Authors:** Haiyu Liu, Sai Zhang, Liang Zhang

**Affiliations:** 1 Department of Neurosurgery, The First Hospital of Jilin University, Jilin, China; 2 Graduate School of Tianjin Medical University, Tianjin, China; 3 Krembil Research Institute, University Health Network, Toronto, ON Canada; 4 Department of Medicine (Neurology), University of Toronto, Toronto, ON Canada

**Keywords:** Artificial cerebrospinal fluid, Epilepsy, Extracellular ion, Ictal-like discharge, Interictal-like spike, Seizure

## Abstract

**Background:**

Rodent brain slices—particularly hippocampal slices—are widely used in experimental investigations of epileptiform activity. Oxygenated artificial cerebrospinal fluid (ACSF) is used to maintain slices in vitro. Physiological or standard ACSF containing 3–3.5 mM K^+^, 1–2 mM Mg^2+^, and 1–3 mM Ca^2+^ generally does not induce population epileptiform activity, which can be induced by ACSF with high K^+^ (8–10 mM), low Mg^2+^, or low Ca^2+^ alone or in combination. While low-Mg^2+^ ACSF without intentionally added Mg salt but with contaminating Mg^2+^ (≤ 50–80 µM) from other salts can induce robust epileptiform activity in slices, it is unclear whether such epileptiform activity can be achieved using ACSF with moderately decreased Mg^2+^. To explore this issue, we examined the effects of moderately modified (m)ACSF with 0.8 mM Mg^2+^, 1.3 mM Ca^2+^, and 5.7 mM K^+^ on induction of epileptiform discharges in mouse hippocampal slices.

**Results:**

Hippocampal slices were prepared from young (21–28 days old), middle-aged (13–14 months old), and aged (24–26 months old) C57/BL6 mice. Conventional thin (0.4 mm) and thick (0.6 mm) slices were obtained using a vibratome and pretreated with mACSF at 35–36 °C for 1 h prior to recordings. During perfusion with mACSF at 35–36 °C, spontaneous or self-sustained epileptiform field potentials following high-frequency stimulation were frequently recorded in slices pretreated with mACSF but not in those without the pretreatment. Seizure-like ictal discharges were more common in thick slices than in thin slices.

**Conclusions:**

Prolonged exposure to mACSF by pretreatment and subsequent perfusion can induce epileptiform field potentials in mouse hippocampal slices.

**Supplementary Information:**

The online version contains supplementary material available at 10.1186/s12868-021-00650-3.

## Scientific statement

Artificial cerebrospinal fluid (ACSF) with high K^+^ (8–10 mM) and/or low Mg^2+^/Ca^2+^ can effectively induce epileptiform activity in rodent brain slices, but this may not fully recapitulate the ionic disturbances observed in vivo under pathologic conditions. Given the moderate-to-severe perturbation of brain interstitial ion homeostasis that occur in neurologic diseases, we examined the effects of moderately modified (m)ACSF with 0.8 mM Mg^2+^, 1.3 mM Ca^2+^, and 5.7 mM K^+^ on induction of epileptiform discharges in mouse hippocampal slices. We found that prolonged exposure to mACSF by pretreatment and subsequent perfusion induced epileptiform field potentials in hippocampal slices of young and adult mice. The protocol described in this study may aid future investigations of hippocampal epileptiform activity in mouse models of neurologic diseases.

## Background

Rodent brain slices—particularly hippocampal slices—are widely used in experimental investigations of epileptiform activity in isolated brain circuits [2, 10, 31]. Oxygenated artificial cerebrospinal fluid (ACSF) is used to maintain slices in vitro. Physiological or standard (s)ACSF contains 3–3.5 mM K^+^, 1–2 mM Mg^2+^, and 1–3 mM Ca^2+^ [10, 31]. Population epileptiform activity is generally not observed in slices perfused with sACSF but can be induced by ACSF containing the K^+^ channel blocker 4-aminopyridine (4-AP), γ-aminobutyric acid (GABA)-A receptor antagonists, or muscarinic receptor agonists [2] or with high K^+^ (8–10 mM), low Mg^2+^, and/or low Ca^2+^ (0–0.2 mM) [10, 31].

Low Mg^2+^ ACSF, which contains no intentionally added Mg salt but has trace amounts of Mg^2+^ (≤ 50–80 µM) as a contaminant from other salts [17, 24] has long been used to induce epileptiform field potentials in slices of the hippocampus or other brain areas [1, 12, 24, 37]. In rat hippocampal slices perfused with low-Mg^2+^ ACSF and monitored using Mg^2+^-selective electrodes, spontaneous epileptiform field potentials started to develop 15–45 min after the onset of perfusion when extracellular Mg^2+^ declined to an estimated concentration of 0.1–0.4 mM [12, 24]. Moreover, perfusion of hippocampi isolated from immature mice with ACSF containing 0.25 mM Mg^2+^, 1.5 mM Ca^2+^, and 5 mM K^+^ induced spontaneous epileptiform field potentials [6–8, 44]. While such low-Mg^2+^ ACSF induced robust epileptiform activity, it may not fully simulate the brain ionic disturbances that occur in pathologic conditions in vivo. Given the moderate-to-severe perturbation of brain ion homeostasis in some neurologic diseases [30], we speculated that moderately modified (m)ACSF could induce hippocampal epileptiform activity. Towards test this hypothesis, in the present study we evaluated the effects of mACSF with 0.8 mM Mg^2+^, 1.3 mM Ca^2+^, and 5.7 mM K^+^ on induction of epileptiform discharges in hippocampal slices of young and adult mice.

## Results

### Slice preparation

Male C57/BL6 mice aged 21–28 days (young), 13–14 months (middle-aged), or 24–26 months (aged) were used for experiments. Brain slices were prepared as previously described [13, 22, 36, 40]. In most experiments, thick slices from young mice were used as they are generally better preserved in vitro than those from adult animals. Briefly, thin (0.4 mm) or thick (0.6 mm) horizontal brain slices encompassing the ventral hippocampal–entorhinal or frontal piriform areas were obtained using a vibratome. In thick slices the dentate gyrus (DG) and CA1 areas were separated along the hippocampal fissure, as we previously showed that such separation allows sufficient tissue oxygenation without disrupting DG–CA3–CA1 connectivity [13, 40]. We focused on the ventral hippocampal circuitry, particularly the CA3 area as it is more susceptible to epileptiform activity than the dorsal hippocampal circuitry [15, 20].

### Slice pretreatment with moderately modified artificial cerebral fluid

Slices were stabilized in a beaker containing sACSF for 45 min and then transferred to another beaker containing mACSF for 1 h. Both sACSF and mACSF were oxygenated with 95% O_2_/5% CO_2_ and maintained at 35–36 °C in a warm bath with automatic temperature control. After the 1-h mACSF exposure, the slices were returned to the sACSF-containing beaker and maintained at room temperature (21–22 °C) for 1–5 h before recordings. Control slices were similarly handled except for the 1-h mACSF exposure. The protocol for exposing slices to mACSF was like that previously used to examine cobalt-induced epileptiform activity in mouse hippocampal slices [13]. For convenience, the 1-h mACSF exposure is hereafter referred to as mACSF pretreatment.

The sACSF was composed of 125 mM NaCl, 3.5 mM KCl, 1.25 mM NaH_2_PO_4_, 2 mM CaCl_2_, 1.3 mM MgSO_4_, 25 mM NaHCO_3_, and 10 mM d-glucose; and mACSF was composed of 129 mM NaCl, 5.7 mM KCl, 1.25 mM NaH_2_PO_4_, 1.3 mM CaCl_2_, 0.8 mM MgSO_4_, 25 mM NaHCO_3_, and 10 mM d-glucose. Both the sACSF and mACSF had a pH of 7.35–7.40 when aerated with 95% O_2_/5% CO_2_.

### In vitro recordings and afferent stimulations

Each thin or thick slice was placed in a submerged recording chamber and perfused with sACSF or mACSF at a perfusate temperature of 35–36 °C. CA3 and CA1 field potentials were simultaneously monitored in most experiments. The data presented herein are for CA3 activity unless otherwise specified. Afferent stimulation was applied to the CA3 area. A single stimulus was used to elicit synaptic field potentials, and high-frequency stimulation (HFS; 100 Hz for 1 s repeated 10 times at a 1-s interval) was used to induce epileptiform field potentials [19]. In most experiments, individual slices were perfused with sACSF or mACSF for ≥ 20 min to detect spontaneous epileptiform field potentials, and HFS was applied if these were absent or if only spontaneous interictal spikes (see below) were observed during the 20-min monitoring period.

### Detection of epileptiform field potentials

**Fig. 1:**
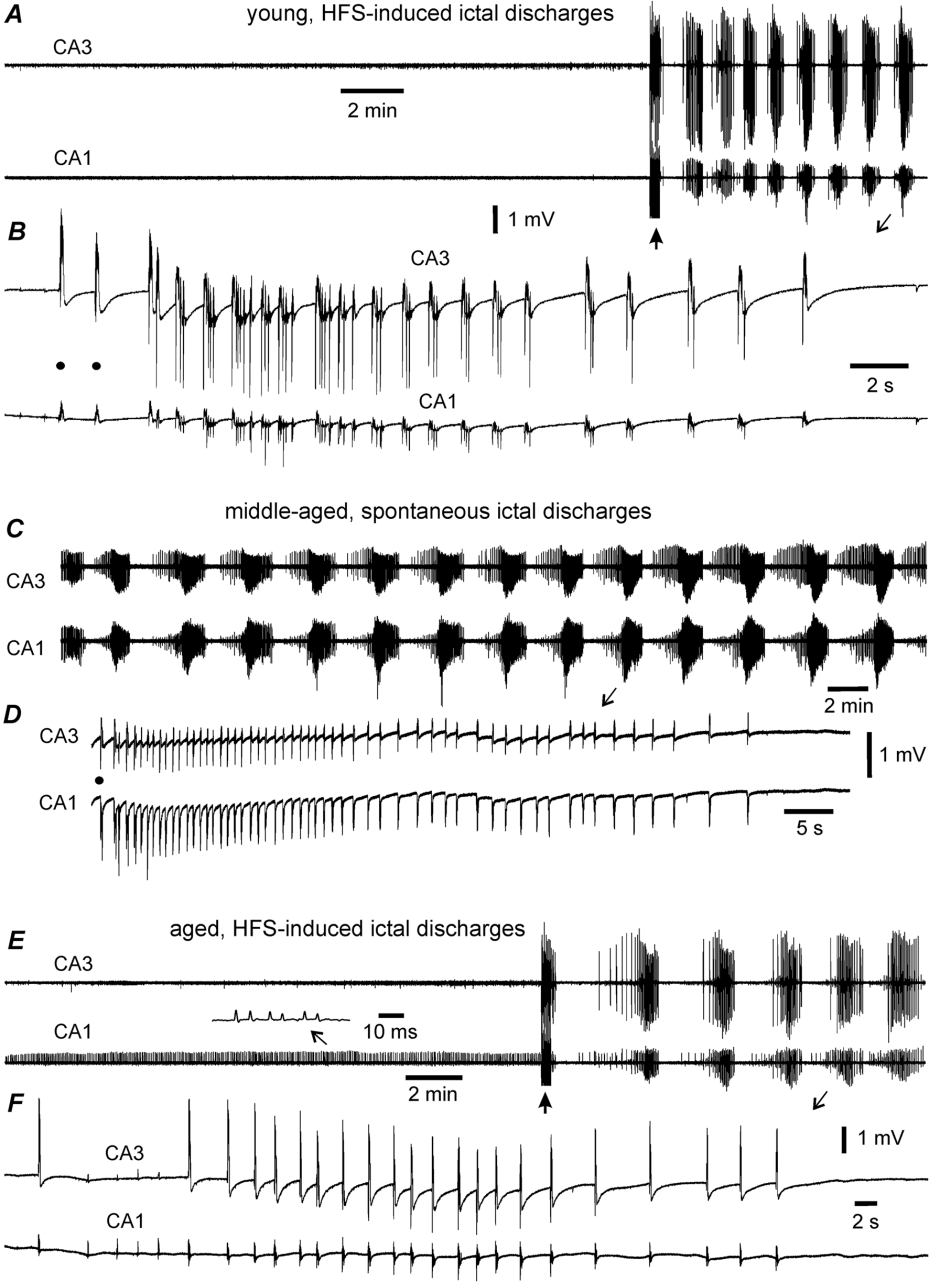
Hippocampal ictal discharges of thin slices. Traces collected from slices of young (**A**, **B**), middle-aged (**D**, **C**) or aged (**E**, **F**) mice. CA3 and CA1 field potentials recorded simultaneously during slice perfusion with moderately modified (m)ACSF. **A** Traces collected before and following high frequency stimulation (HFS, filled arrow) and illustrated after treatment with a band-pass filter (2–500 Hz). **B** A discharge event expanded in a wide frequency band (0–1000 Hz). Filled circles denoted preictal spikes. **C**–**F** Spontaneous or HFS-induced ictal discharges similarly illustrated as above. CA3 unit spikes expanded (**E**, insert). Note discharges with the hypersynchronous onset in **B** and **D** and discharges without an evident component of the hypersynchronous onset in **F**

**Fig. 2:**
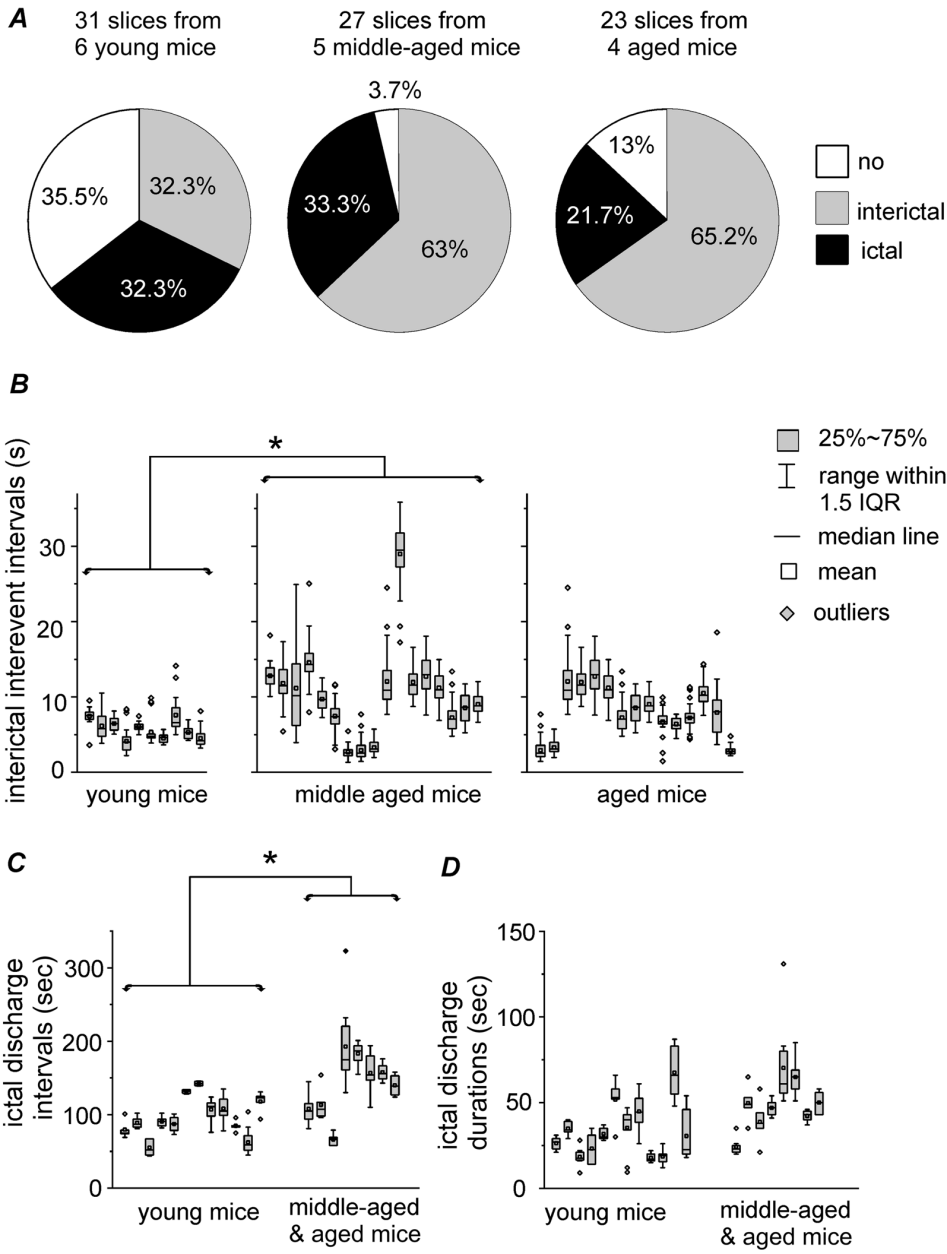
Measures for hippocampal ictal and interictal events of thin slices. Slices obtained from young (21–28 days old), middle-aged (13–14 months old) and aged (24–26 months old) mice. **A** Proportions of slices with or without epileptiform field potentials estimated from the three age groups. The “ictal” category refers to slices that displayed discharges spontaneously or following high frequency stimulation (HFS), irrespective of associated interictal spikes; the “interictal” refers to slices that expressed interictal spikes spontaneously or following HFS, but no spontaneous or HFS-induced ictal discharges; the “no” denotes slices that did not exhibit spontaneous nor HFS-induced epileptiform field potentials. **B** Interevent intervals of hippocampal interictal spikes measured in individual slices of the three age groups. **C**, **D** Interevent intervals and durations of ictal discharges measured in individual slices with ≥ 5 ictal events. Measures for slices of the middle-aged and aged groups were pooled. Box plots presented **B**–**D**, and interquartile range abbreviated as IQR. *One-way analysis of variance (**B**) or Student t test (**C**), p < 0.05, median values comparison

**Fig. 3:**
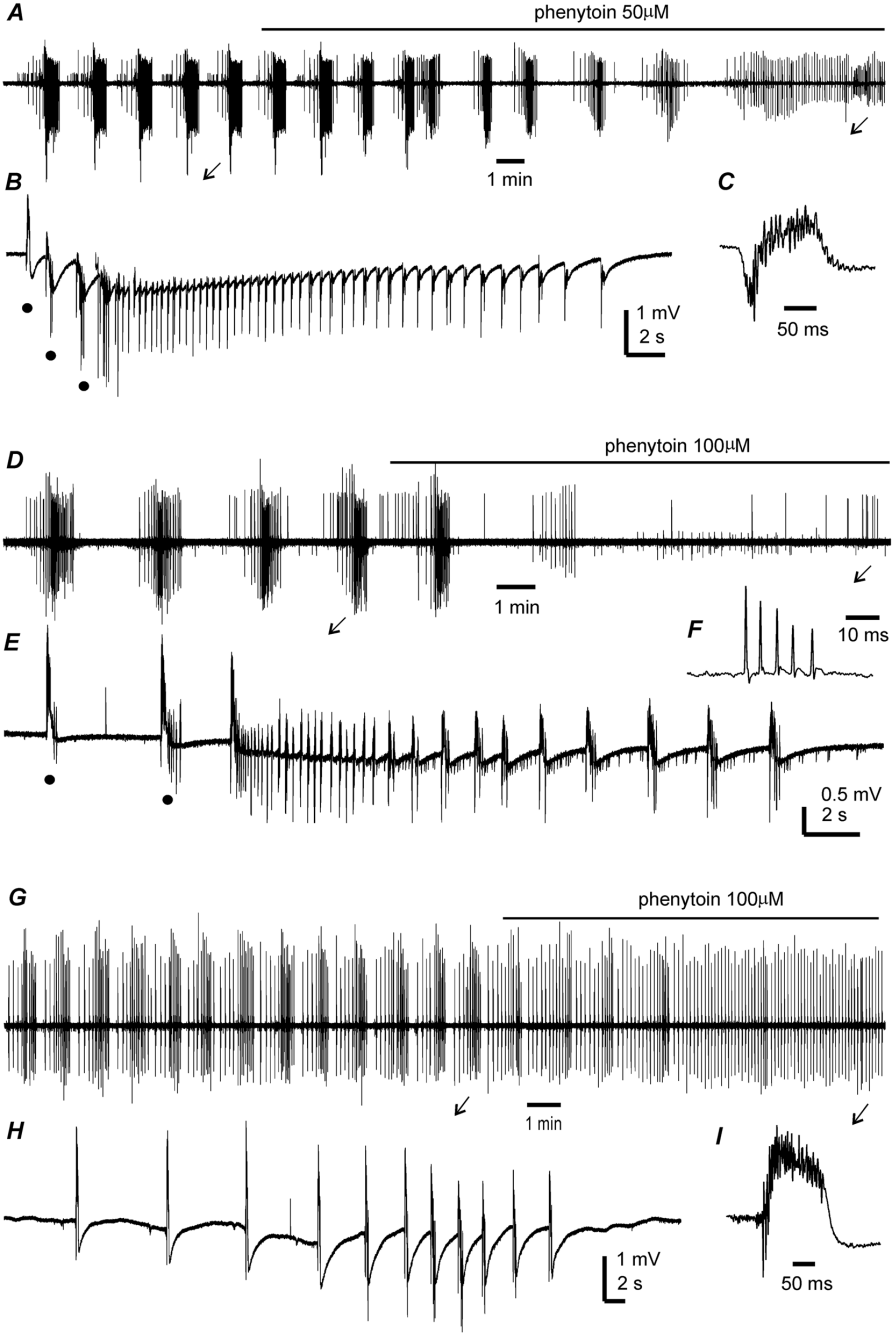
Effects of phenytoin on hippocampal epileptiform activities. Extracellular field potentials recorded from two thick slices (**A**–**E**) and one thin slice (**G**–**I**) of young mice. Spontaneous (**A**–**E**) or high frequency stimulation (HFS)-induced discharge (**G**–**I**) collected during slice perfusion with moderately modified (m)ACSF. Phenytoin added in mACSF at indicated concentrations and application times denoted by solid lines above traces. **A** Spontaneous CA3 field potentials recorded before and following phenytoin application and illustrated after treatment with a band-pass filter (2–500 Hz). **B**, **C** arrowed events expanded in a wide frequency band (0–1000 Hz). Filled circles in B denote preictal spikes. **D–F** and **G–I**, CA3 field potentials similarly illustrated as above. Note discharges with the hypersynchronous onset in **B** and **E** and the discharge without an evident component of the hypersynchronous onset in **H**

**Fig. 4:**
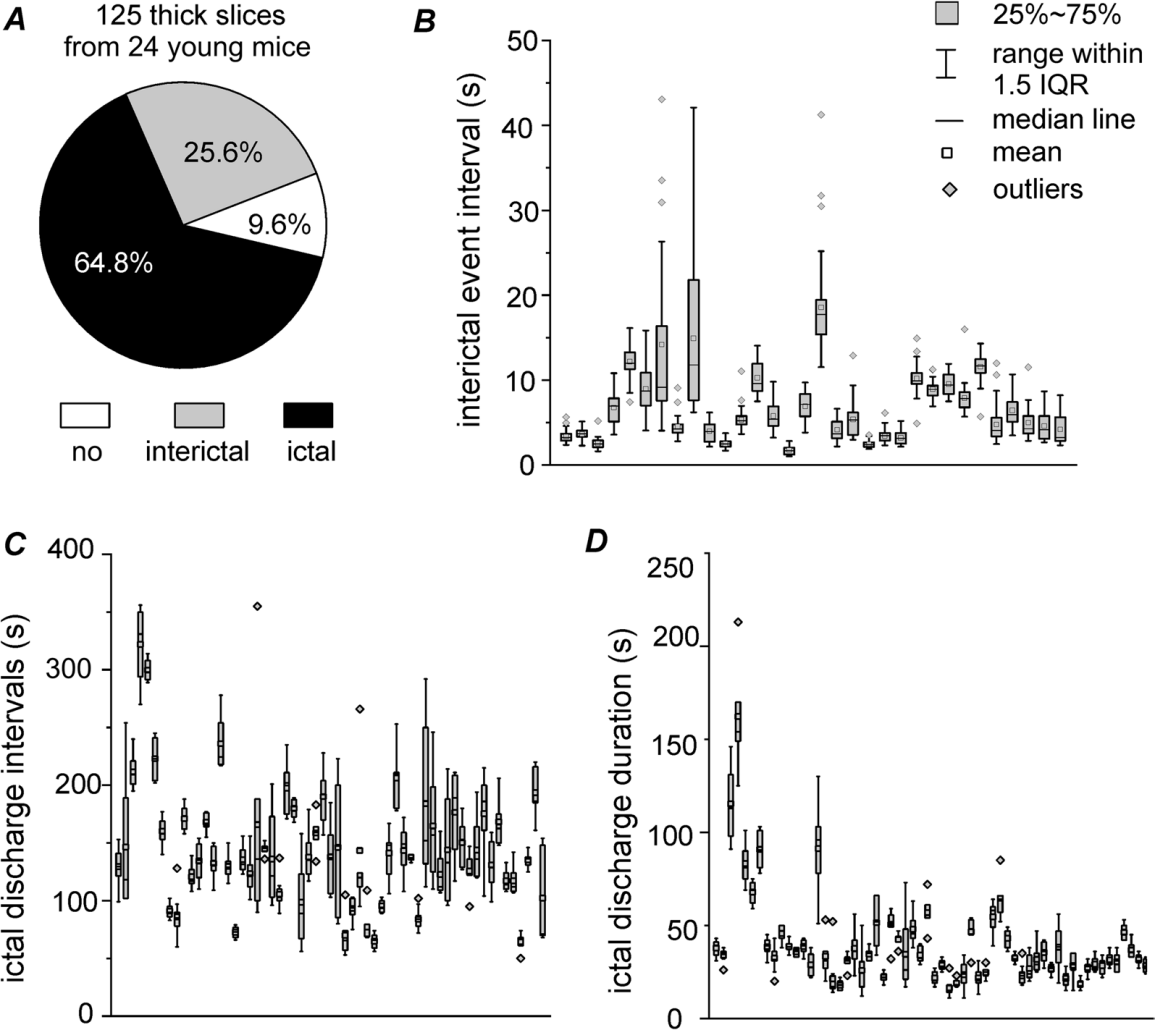
Measures for hippocampal ictal and interictal events of thick slices. **A** Thick hippocampal slices obtained from young mice (21–28 days-old). Proportion of slices with or without detected ictal discharges or interictal spikes was estimated. The “ictal” category refers to slices that displayed discharges spontaneously or following HFS, irrespective of associated interictal spikes; the “interictal” refers to slices that expressed interictal spikes spontaneously or following HFS, but no spontaneous or HFS-induced ictal discharges; the “no” denotes slices that did not exhibit spontaneous nor HFS-induced epileptiform field potentials. **B** Interevent intervals of interictal spikes measured from individual thick slices. **C**, **D** Interevent intervals and durations of ictal discharges measured from individual slices with ≥ 5 recurrent discharge events. Box plots presented in **B**–**D**, and interquartile range abbreviated as IQR

**Fig. 5:**
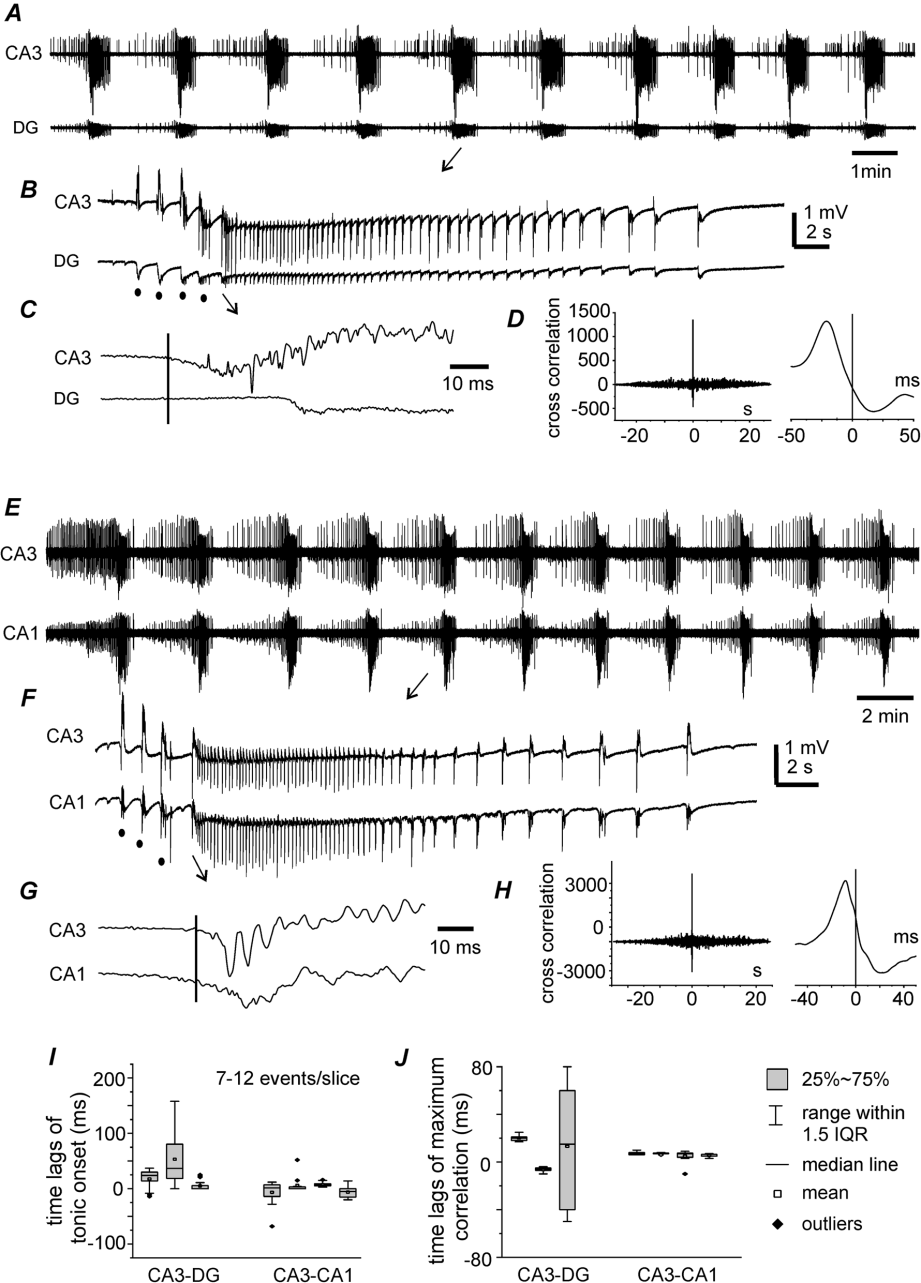
Temporal relation of hippocampal regional discharges. Data collected from thick slices of young mice (7–12 ictal events per slice). All traces collected during slice perfusion with moderately modified ACSF. **A** Continuous traces collected simultaneously from CA3 and dentate gyrus (DG) areas and illustrated after treatment with a band-pass filter (2–500 Hz). **B**, **C** Arrowed events expanded in a wide frequency band (0–1000 Hz). Filled cycles denoted preictal spikes. A solid vertical line denoted the putative onset of CA3 discharge. **D** A cross correlation plot generated from the discharges in **B**. X axis of the plot expanded at right. Note CA3 preceding DG signals by ~ 25 ms in the time lag of maximum correlation. **E**–**H**, CA3 and CA1 discharges similarly illustrated and analyzed as above. Note CA3 preceding CA1 signals by ~ 10 ms in the time lag of maximum correlation. **I** Onset time lags measured from corresponding CA3-DG (3 slices) or CA3-CA1 discharges (4 slices). **J** Time lags of maximum correlation in cross correlation plots. Positive measures refer CA3 preceding DG or CA1 signals. Box plots presented in **I**, **J**, and interquartile range abbreviated as IQR

**Fig. 6:**
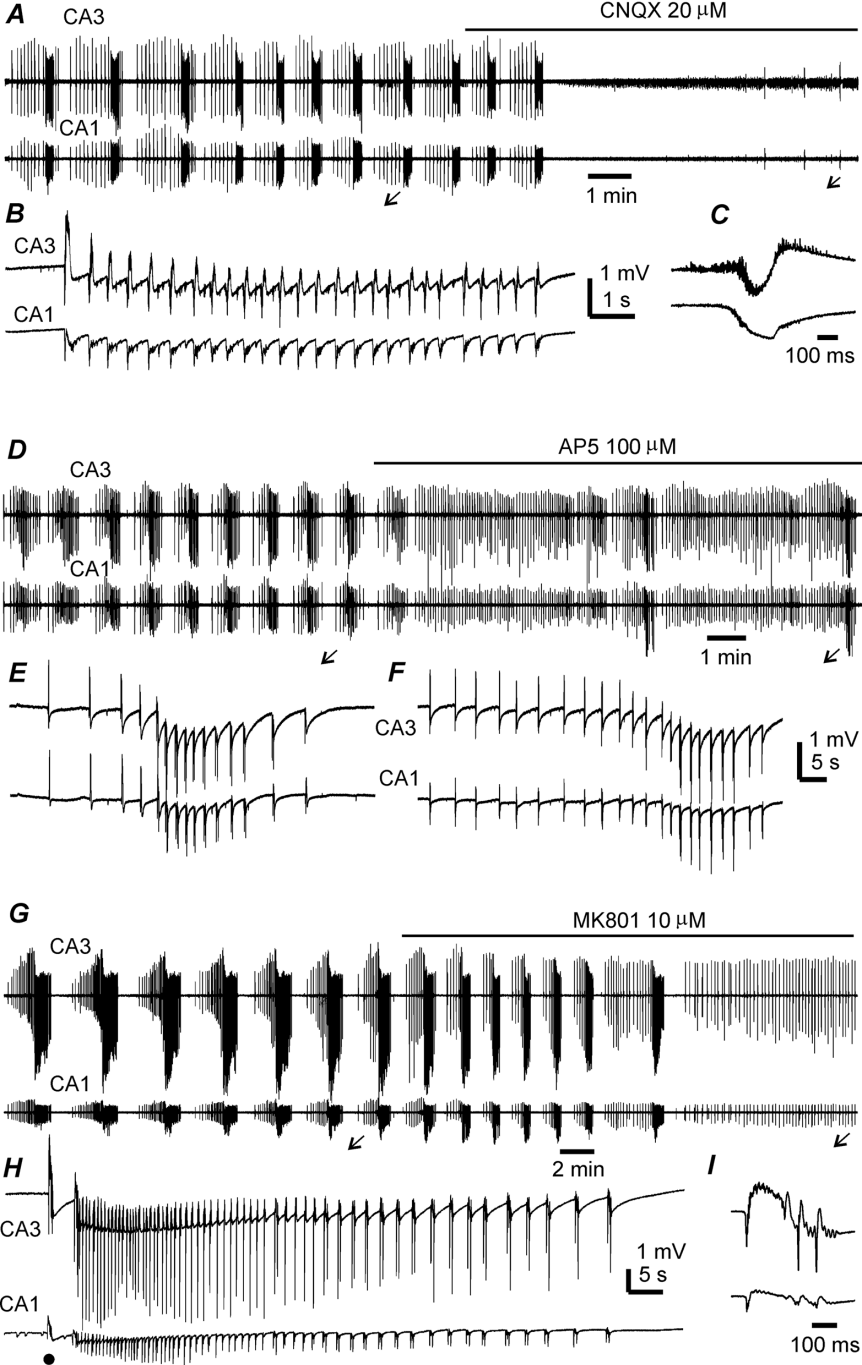
Effects of glutamate receptor antagonists on hippocampal epileptiform activates. Extracellular field potentials recorded from three thick slices of two young mice. Traces collected following slice perfusion with moderately modified (m)ACSF. CNQX, AP5 and MK8-1 added in mACSF at indicated concentrations, and application times indicated by solid lines above traces. **A** Field potentials recorded before and following CNQX application and illustrated after treatment with a band-pass filter (2–500 Hz). **B**, **C** Arrowed events expanded in a wide frequency band (0–1000 Hz). **D**–**F** and **G**–**I** Field potentials recorded before and following AP5 or Mk801 application and similarly illustrated as above. Note discharges with the hypersynchronous onset in **B** and **H** and discharges without an evident component of the hypersynchronous onset in **E**, **F**

**Fig. 7:**
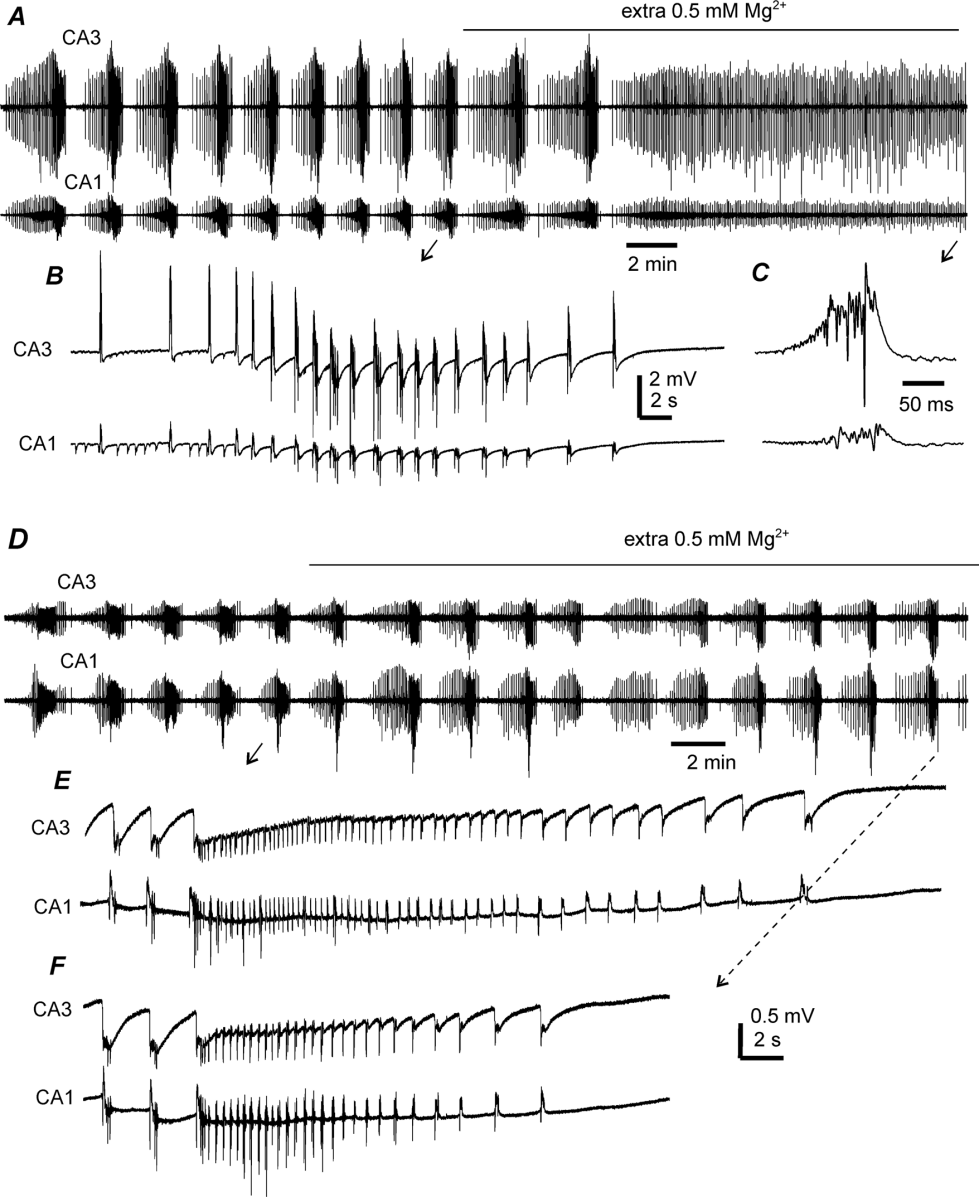
Effects of adding extra 0.5 mM Mg^2+^ on hippocampal epileptiform activities. Data collected from two thick hippocampal slices of two young mice. Extracellular filed potentials recorded simultaneously from CA3 and CA1 areas and during perfusion with moderately modified (m)ACSF. Extra 0.5 mM Mg^2+^ added in mACSF and application times denoted by solid lines above traces. Original signals in A and D illustrated after treatment with a band-pass filter (2–500 Hz). Arrowed events in **B**, **C** and **E**, **F** expanded in a wide frequency band (0–1000 Hz). Note that adding extra 0.5 mM Mg^2+^ in mACSF suppressed ictal discharges (**A**, **C**) or reduced discharge durations (**D**, **F**)

Two types of mACSF-induced epileptiform field potentials were observed—namely, ictal-like discharges and interictal-like spikes—that were like those induced by low-Mg^2+^ ACSF in rat hippocampal slices [1, 12, 24, 37] and mouse hippocampi [6–8, 44]. Specifically, the ictal discharges comprised repetitive spike waveforms with a duration ≥ 10 s and recurring at an interevent interval of ≥ 40 s. Discharge waveforms were predominantly positive (upward) or negative (downward) when recorded from CA3 and CA1 somatic and apical dendritic layers, respectively. Most ictal discharges showed a hypersynchronous onset [10] with a few pre-ictal spikes. The occurrence of discharges was associated with a negative baseline shift by approximately 0.5 mV. The termination of discharges was followed by a postictal suppression phase of variable length that clearly separated recurrent discharges. Examples of ictal discharges recorded from thin hippocampal slices are illustrated in Fig. 1; measurements of epileptiform field potentials from thin slices are presented in Fig. 2. Examples of ictal discharges observed from thick hippocampal slices are shown in Fig. 3; measurements of epileptiform field potentials from thick slices are presented in Fig. 4. Ictal discharges and interictal spikes recorded from thick hippocampal slices are also shown in Figs. 5, 6, 7.

The interictal spikes featured periodic events with a base duration of 50–300 ms and incidence of 2–7 events/10 s (Figs. 2B and 4B) and had similar waveform polarity as ictal discharges (i.e., with positive or negative waveforms when recorded from CA3 and CA1 somatic and epical dendritic areas, respectively). The somatic interictal spikes were often superimposed with ripple-like oscillatory activity of 200–250 Hz (Additional file 1: Fig. S1). Single- or multi-unit spike activity with a base duration ≤ 5 ms and variable amplitude and spikes rate (Additional file 2: Fig. S2) was not considered as an epileptiform field potential because it was not uniformly detected in the target area.

### Initial exploration of the effects of moderately modified artificial cerebral fluid

To test whether mACSF perfusion induced epileptiform field potentials in control slices (i.e., without pretreatment), thin slices from young mice were perfused with mACSF for ≥ 60 min. Extracellular recordings were made from the CA3 and CA1 areas before and following mACSF perfusion. In six slices obtained from three mice, no spontaneous or HFS-induced epileptiform field potential was observed following mACSF perfusion; and CA3 population spikes did not differ significantly in peak amplitude when evoked before and following mACSF perfusion for ≥ 60 min (0.9 ± 0.2 vs 1.4 ± 0.5 mV), although double spikes were noted in three of the six slices following mACSF perfusion.

To determine whether mACSF perfusion induced epileptiform field potentials in mACSF-pretreated slices, thin slices from young mice were pretreated with mACSF for 1 h prior to recording. Each slice was initially perfused with sACSF for about 20 min and then with mACSF. In 14 pretreated slices from three mice, no spontaneous epileptiform field potentials were observed during the first 20 min of sACSF perfusion but emerged in 10/14 slices after several minutes of mACSF perfusion. Specifically, 2/10 slices showed spontaneous ictal discharges and the other 8 showed spontaneous interictal-like spikes only (Additional file 2: Fig. S2). These results suggest that prolonged exposure of slices to mACSF by pretreatment and subsequent perfusion was more effective than perfusion alone to induce epileptiform activity in mouse hippocampal slices. Therefore, in subsequent experiments, slices were pretreated and then directly perfused with mACSF to induce epileptiform field potentials.

### Hippocampal epileptiform potentials in thin slices from young and adult mice

The emergence of epileptiform activity in vitro is strongly affected by the age of the animal from which the brain slice is derived [31]. To explore this issue in our model, thin slices from young, middle-aged, and aged mice were pretreated with mACSF for 1 h and perfused with mACSF for ~ 20 min during the recording to monitor spontaneous epileptiform field potentials; if these were absent or if only spontaneous interictal spikes were observed during the 20-min period, HFS was applied. Ictal discharges and interictal spikes in slices showed similar waveforms in the three age groups (Fig. 1). Additionally, once stabilized, HFS-induced ictal discharges and interictal spikes were comparable to spontaneous events in terms of waveform and frequency. Therefore, slices with spontaneous or HFS-induced ictal or interictal events in each age group were pooled (Fig. 2A). Slices that developed ictal discharges either spontaneously or following HFS irrespective of associated interictal spikes were considered as ictal expressing slices; those with interictal spikes but not ictal discharges that developed spontaneously or following HFS were considered as interictal expressing slices; and those that showed no spontaneous or HFS-induced epileptiform field potentials were considered as having no epileptiform activity.

In 31 thin slices from six young mice, spontaneous epileptiform field potentials were not detected during the 20-min initial monitoring period; self-sustained epileptiform field potentials were observed following HFS in 20 of the 31 slices, with expression of ictal discharges in 10 slices and interictal spikes in the other 10 (Fig. 2A, left). In 27 thin slices from five middle-aged mice, spontaneous or HFS-induced epileptiform field potentials were observed in 26 slices. Specifically, spontaneous ictal discharges occurred in two slices and were self-sustaining following HFS in seven slices; and interictal spikes occurred spontaneously or following HFS in 17 slices (Fig. 2A, middle). In 23 thin slices from four aged mice, spontaneous or HFS-induced epileptiform potentials were observed in 20 slices. Specifically, ictal discharges arose spontaneously in one slice and were self-sustaining following HFS in four slices; and spontaneous or HFS-induced interictal spikes were recorded in 15 slices (Fig. 2A, right).

The proportion of slices with spontaneous and HFS-induced epileptiform field potentials (ictal plus interictal events) did not differ significantly across the three age groups (Fig. 2A). However, slices from young mice showed interictal spikes (Fig. 2B) and ictal discharges (Fig. 2C) with a shorter interevent interval compared to those from middle-aged mice and aged mice, respectively.

HFS-induced epileptiform field potentials often appeared within 3 min post HFS (Fig. 1A, B). The time until the emergence of spontaneous epileptiform field potentials in individual slices could not be determined in the above experiments because each slice was directly perfused with mACSF once it was placed in the recording chamber and the placement of stimulating and recording electrodes and assessment of evoked responses took several minutes. Spontaneous epileptiform field potentials were observed at the beginning of continuous extracellular recording or gradually emerged in the first 5–8 min (Fig. 1C, D).

### Epileptiform field potentials in piriform and entorhinal areas

Thin slices encompassing the piriform or entorhinal areas obtained from young mice were pretreated with mACSF. In 2 or 3 of the 17 piriform slices from three mice, spontaneous or HFS-induced ictal discharges were observed following mACSF perfusion (Additional file 3: Fig. S3). These discharges had a mean duration of 40.2 ± 3.3 s and interevent interval of 145.3 ± 5.8 s (31 events/5 slices). In the remaining 12 piriform slices, no mACSF-induced epileptiform field potentials were observed before or after HFS. Spontaneous and HFS-induced entorhinal interictal events, but not ictal discharges, were observed in 4 of 10 entorhinal slices from three young mice. These interictal spikes had a mean interevent interval of 57.0 ± 8.9 s (26 events/4 slices). These results indicate that mACSF-induced epileptiform activity was detectable to varying degrees in seizure-prone forebrain circuits other than the hippocampus [39].

### Epileptiform activity observed in thick hippocampal slices of young mice

In our previous work, spontaneous epileptiform field potentials were more frequently observed in thick as compared to thin mouse hippocampal slices [13]. We therefore tested whether the same was true for mACSF-induced epileptiform activity. Thick slices (0.6 mm) of the ventral hippocampus from young mice were pretreated and perfused with mACSF during recording. The hippocampal ictal and interictal spike waveform in thick slices (Figs. 3, 5, 6, 7) was comparable to that in thin slices.

mACSF-induced epileptiform field potentials were observed in 107 of 125 thick hippocampal slices from 24 young mice, including ictal discharges in 81 slices and interictal spikes in 26 (Fig. 4A–D). The observed ictal discharges were either spontaneous (64 slices) or induced by HFS (17 slices). Most discharges showed a hypersynchronous onset with pre-ictal spikes [10] (Figs. 5B, E, 6B, 7G. Of the 71 thick slices with relatively stable recurrent ictal discharges (≥ 5 events/slice), 45 showed discharges with a hypersynchronous onset; 19 had discharges without an obvious hypersynchronous onset; and 7 had a mixed onset discharge pattern.

The overall rate of ictal discharges was significantly higher in thick slices than in thin slices from young mice (81/117 vs 10/31 slices, p = 0.0003). In slices with ≥ 5 recurrent ictal discharges, the interevent interval was longer in thick slices (Fig. 4C) than in thin slices (Fig. 2C) (median value comparison, p = 0.0043), whereas ictal discharge duration did not differ significantly between the two groups (Figs. 2D and 4D). These observations suggest that preservation of a relatively extensive mouse hippocampal circuitry promotes the development of ictal-like discharges in vitro. As ictal discharges were more consistently observed in thick slices than in thin ones, we used the former in most subsequent experiments.

### Suppression of hippocampal ictal discharges by phenytoin

The effects of the antiepileptic drug phenytoin were examined in seven hippocampal slices obtained from five mice. Phenytoin was added to mACSF at a concentration of 50 or 100 µM and applied for 8–10 min. Spontaneous discharges were suppressed by application of 50 or 100 µM phenytoin in three thick slices from two young mice (Fig. 4A–E); HFS-induced discharges were suppressed or shortened by 100 µM phenytoin in three thin slices from two young mice (Fig. 4G, H); and application of 50 µM phenytoin reduced discharge duration in one thin slice from a middle-aged mouse. While phenytoin suppressed or decreased the duration of ictal discharges, interictal spikes (Fig. 4A, C, G, I) or unit spike activity (Fig. 4D, F) remained detectable. This is in line with the in vivo effects of phenytoin in kindled mice observed via intra-hippocampal electroencephalographic recordings [35]. Other clinically used antiepileptic drugs were shown to abolish ictal discharges but not interictal events induced by 4-AP in rat entorhinal slices [5]. We speculate that mACSF-induced ictal discharges present as seizure-like events in mouse hippocampal slices.

### Ictal discharges observed in hippocampal subregions

Discharges in different hippocampal regions were examined in seven thick slices from four young mice. Specifically, mACSF-induced spontaneous discharges with hypersynchronous onset were simultaneously recorded from the CA3 and DG areas in three thick slices (12–16 events/slice). The onset times of corresponding regional discharges and cross correlation of discharge signals were assessed. Discharges in the CA3 area preceded those in the DG by 5.0 ± 2.3 to 17.6 ± 4.5 ms (Fig. 5A–C). Corresponding CA3 and DG discharges were highly correlated (Fig. 5D), but the time lags of the maximum correlation between CA3 and DG discharge signals varied (Fig. 5J).

mACSF-induced CA3 and CA1 discharges were recorded from four thick slices (7–14 events/slice) (Fig. 5E–H). CA3 discharges preceded CA1 discharges by 2.4 ± 3.5 to 10.8 ± 1.6 ms in three slices and succeeded CA1 discharges by 4.4 ± 4.4 ms in one slice (Fig. 5I). Corresponding CA3 and CA1 discharge signals were highly correlated, and CA3 signals consistently preceded those in the CA1 area by 4.9 ± 1.8 to 7.1 ± 0.4 ms in terms of time lags of the maximum correlation (Fig. 5J). These observations suggest that mACSF-induced ictal discharges are mainly initiated in the CA3 area. The Schaffer collateral pathway conveys discharge signals that spread from CA3 to CA1, whereas the mossy fiber pathway and DG-to-CA3 back-projection [27, 33] may determine or influence the temporal relationship between CA3 and DG discharge signals.

### Effect of glutamate receptor antagonist on hippocampal ictal discharges

The effects of the α-amino-3-hydroxy-5-methyl-4-isoxazolepropionic acid (AMPA) receptor antagonist 6-cyano-7-nitroquinoxaline-2,3-dione (CNQX; 20 µM) and N-methyl-d-aspartate (NMDA) receptor antagonists 2-amino-5-phosphonopentanoate (AP5) (100 µM) and MK801 (10 µM) were examined in 21 thick slices from five young mice. In five slices with baseline ictal discharges of 5.2 ± 0.73 events/10 min, no discharge was observed following bath application of 20 µM CNQX for 5–8 min, although a few sporadic events with a small amplitude were noted (Fig. 6A–C). CNQX application similarly abolished CA3 and CA1 interictal spikes but not unit activity in all four examined slices.

Before AP5 application, CA3 discharges in four slices had a mean interevent interval of 95.9 ± 8.1 s and mean duration of 28.9 ± 3.2 s (5–10 events/slice). Bath application of 100 µM AP5 for 10–15 min suppressed ictal discharges in three slices and attenuated discharges in one slice (Fig. 6D–F). Before MK801 application, CA3 ictal discharges in eight slices had an interevent interval of 129.2 ± 5.7 s and duration of 40.7 ± 1.8 s; these were abolished in seven slices and greatly attenuated in the one remaining slice by bath application of 10 µM MK801 for 10–15 min (Fig. 6G, H). While ictal discharges were suppressed or attenuated by AP5 or MK801, interictal spikes remained detectable in the CA3 and CA1 areas (Fig. 6F, I). These results suggest that the activity mediated by AMPA receptors is essential for the generation of both ictal discharges and interictal spikes, whereas aberrant NMDA receptor activation plays an important role in sustaining ictal discharges but not interictal spikes.

### Effect of additional Mg^2+^ on hippocampal epileptiform field potentials

The effects of supplementing mACSF with 0.5 mM Mg^2+^ (or increasing Mg^2+^ concentration from 0.8 to 1.3 mM) were examined in seven thick hippocampal slices from two young mice. CA3 discharges recorded during perfusion with mACSF (containing 0.8 mM Mg^2+^) had an interevent interval of 102.6 ± 4.6 s and duration of 39.7 ± 2.9 s (n = 48 events/7 slices, 5–12 ictal events/slice). Discharges were suppressed in four of the seven slices following supplementation with 0.5 mM Mg^2+^ for 5–10 min, while CA3 and CA1 interictal spikes remained detectable (Fig. 7A–C). CA3 and CA1 ictal discharges persisted in the other three slices following application of additional Mg^2+^ for 15–20 min, but the discharge duration was significantly reduced (30.2 ± 1.4 s [n = 18 events] and 19.0 ± 1.4 s [n = 14 events] in the presence of 0.8 and 1.3 mM Mg^2+^, respectively; p < 0.001; Fig. 7D–F). Overall, the effects of Mg^2+^-supplemented mACSF on ictal discharges and interictal spikes were comparable to those induced by application of AP5 or MK801.

### Discussion

The primary goal of our study was to investigate whether mACSF with 0.8 mM Mg^2+^, 1.3 mM Ca^2+^, and 5.7 mM K^+^ could induce epileptiform activity in mouse hippocampal slices. Instead of monitoring individual slices during prolonged mACSF perfusion, slices were pretreated with mACSF for 1 h and then perfused with mACSF during recording. This protocol allowed multiple slices to be exposed to mACSF in a relatively consistent manner and facilitated the induction of epileptiform activity during recording. mACSF-pretreated hippocampal slices had a high propensity to exhibit epileptiform activity either spontaneously or after HFS, and the rates of epileptiform field potentials were similar in thin slices from young, middle-aged, and aged mice. These observations indicate that pretreatment and subsequent perfusion with mACSF can induce epileptiform activity in mouse hippocampal slices. mACSF-induced epileptiform activity was also observed in the piriform and entorhinal cortical areas, suggesting that the mACSF application protocol may be suitable for investigating epileptiform activity in mouse brain slices harboring other seizure-prone neural circuits [39].

Both mACSF-induced ictal discharges and interictal spikes were abolished by application of the AMPA receptor antagonist CNQX. Ictal discharges were also suppressed or attenuated while interictal spikes remained detectable following application of the NMDA receptor antagonist AP5 or MK801; and ictal discharges but not interictal spikes were suppressed or attenuated by mACSF supplemented with 0.5 mM Mg^2+^. These results imply that the generation of mACSF-induced epileptiform activity is dependent on glutamatergic hyperexcitation, and that aberrant NMDA receptor activation plays an important role in sustaining ictal discharges, which is in keeping with earlier work in rat hippocampal slices [1, 12, 21, 24, 37] and immature mouse hippocampi [6–8, 44]. Furthermore, mACSF-induced ictal discharges but not interictal spikes were sensitive to inhibition by the antiepileptic drug phenytoin. This is consistent with the finding that other clinically used antiepileptic drugs (carbamazepine, topiramate, and valproic acid) abolished ictal discharges but not interictal events induced by 4-AP in rat entorhinal slices [5]. Notwithstanding the pretreatment protocol used in our experiments, hippocampal epileptiform activity induced by mACSF appears to have neurochemical and pharmacologic properties that are like those described in previous studies [1, 5–8, 12, 21, 24, 37, 44].

In rat hippocampal slices perfused with low-Mg^2+^ ACSF (≤ 50–80 µM as a contaminant from other salts), spontaneous epileptiform field potentials started to develop 15–45 min after the onset of ACSF perfusion [24]. In hippocampi isolated from immature mice (6–25 days old) and perfused with ACSF containing 0.25 mM Mg^2+^, 1 mM Ca^2+^, and 5 mM K^+^, spontaneous ictal discharges were observed following 15–45 min of ACSF perfusion [8]. In the present study, spontaneous or HFS-induced epileptiform field potentials were not detected in control slices (i.e., without mACSF pretreatment) following mACSF perfusion for ≥ 60 min; however, in mACSF-pretreated slices, spontaneous epileptiform field potentials were observed within 10 min of mACSF perfusion. The mACSF exposure time (pretreatment plus subsequent perfusion) required to induce epileptiform field potentials in these pretreated slices was ≥ 65 min, which is considerably longer than the time reported for rat hippocampal slices [24] and immature mouse hippocampi [8]. This difference may be attributable to multiple experimental factors including the ionic components of ACSF. In particular, the low-Mg^2+^ ACSF (with 0.25 mM Mg^2+^, 1 mM Ca^2+^, and 5 mM K^+^) used in previous investigations of immature mouse hippocampi [6–8, 44] and the mACSF (with 0.8 mM Mg^2+^, 1.3 mM Ca^2+^, and 5.7 mM K^+^) used in our study differed slightly in terms of K^+^ and Ca^2+^ concentrations (by 0.7 and 0.2 mM, respectively), but Mg^2+^ concentration was threefold higher in our formulation. As we used the same mouse strain (C57BL/6) and similar recording conditions (submerged recording chambers with fast perfusion) as in earlier work [7, 8], 2008), an age-related decline in susceptibility to induced epileptiform activity and higher Mg^2+^ concentration in mACSF could partly explain why prolonged mACSF exposure was required to induce epileptiform field potentials in hippocampal slices from young and adult mice. It is also conceivable that mACSF with 0.8 mM Mg^2+^ moderately affects Mg^2+^-dependent blockade of NMDA receptor [23, 26] and the surface charge screening of divalent cations [16] compared to ACSF containing ≤ 50–80 µM Mg^2+^ [24] or 0.25 mM Mg^2+^ [8].

mACSF-induced ictal discharges were observed in 32%, 33%, and 22% of thin hippocampal slices from young, middle-aged, and aged mice, respectively; most of these were induced by HFS. The rate of ictal discharge was greatly increased in thick hippocampal slices from young mice; discharges were observed in 65% of thick slices and most of these occurred spontaneously. This is in line with the results of a previous study, in which spontaneous ictal discharges were recorded in 74% of immature mouse hippocampi following perfusion with low-Mg^2+^ (0.25 mM) ACSF [8]. Additionally, cobalt-induced ictal discharges were more frequently observed in thick as compared to thin mouse hippocampal slices [13]. Collectively, our observations and evidence from earlier reports suggest that preservation of a relatively large proportion of hippocampal circuitry promotes the generation of seizure-like ictal discharges in vitro.

One concern regarding our experimental design is that after vibratome sectioning, hippocampal slices were allowed a 45-min recovery period prior to mACSF pretreatment. Preparation of brain or hippocampal slices is known to be associated with ischemic/hypoxic and traumatic perturbations, which affect energy metabolism and the functionality of slice tissues. In hippocampal slices prepared from young rats (17–27 days old), tissue adenine nucleotide level was found to stabilize after 3 h of incubation in sACSF at 34 °C [46]. It is therefore highly likely that in our experiments, mouse hippocampal slices were metabolically unstable prior to mACSF pretreatment. Dissection-related changes in adenine nucleotides and other relevant metabolites together with the 1-h mACSF pretreatment may have elicited a state of heightened hyperexcitability in the slices. This issue could be addressed in future experiments using the mACSF pretreatment protocol with a longer recovery period after vibratome sectioning. Additionally, the effects of other clinically used antiepileptic drugs and neurotransmitter receptor antagonists on mACSF-induced ictal discharges need to be evaluated, and the cellular mechanisms and local circuitry that underlie the emergence of mACSF-induced epileptiform field potentials in mouse hippocampal slices remain to be elucidated. Despite these limitations, the protocol described in our study provides a basis for future investigations of mACSF-induced epileptiform field potentials and especially seizure-like ictal discharges.

The pathophysiologic significance of our in vitro observations is unclear. Mg^2+^ concentration in cerebrospinal fluid was found to be decreased by ~ 0.3 mM in patients with acute ischemic stroke [3], additionally, cortical Mg^2+^ was decreased by ~ 0.2 mM [38] and hippocampal extracellular K^+^ was increased one–twofold [18] in rat models of moderate fluid percussion brain injury. Ischemic or traumatic brain injury is associated with acute seizures [4, 28, 29], which may be caused by perturbation of brain interstitial ion homeostasis [30]. Based on these findings, we speculate that mACSF simulates moderate ionic perturbations associated with brain ischemic/traumatic injury and that our in vitro model of mACSF-induced epileptiform activity can be useful for exploring the pathophysiologic mechanisms underlying the development of acute seizures following such injury.

## Conclusion

We examined the effects of mACSF with 0.8 mM Mg^2+^, 1.3 mM Ca^2+^, and 5.7 mM K^+^ on induction of epileptiform discharges in mouse hippocampal slices. We demonstrated that prolonged slice exposure to mACSF via a pretreatment and subsequent perfusion protocol induced epileptiform field potentials in slices from young and adult mice, and that seizure-like ictal discharges were more prominent in thick slices than in conventional thin hippocampal slices. Given the moderate-to-severe perturbation of brain interstitial ion homeostasis in neurologic diseases, the protocol described herein may aid future investigations of hippocampal epileptiform activity in mouse models of neurologic diseases.

## Methods

### Animals

C57BL/6 mice, which are widely used in neuroscience and genetic/molecular studies, were used in our experiments. C57 mice can have a lifespan of up to 30 months [11] and their ages of 3–5, 16–18, and 24–28 months are thought to correspond to human ages of 20–30, 51–56, and 69–73 years, respectively [11]. The mice used for our in vitro experiments were 21–28 days, 13–14 months, or 24–26 months old and are referred to as young, middle-aged, and aged, respectively.

The mice were obtained from Charles River Laboratories (St-Constant, QC, Canada). Male mice were used in the present study to avoid potential influences of sex hormones on hyperexcitability and induced epileptiform activity [34]. All experiments conducted in this study were reviewed and approved by a local animal care committee per the Guidelines of the Canadian Council on Animal Care (University Health Network Animal Use Protocol #986.40). Mice were housed 3–4 per cage with free access to food and water in a local vivarium that was maintained at a temperature of 22–23 °C on a 12:12-h light/dark cycle (lights on starting at 6:00 am). Each cage had a plastic cylinder tube and a few pieces of cotton for nesting.

### Slice preparation

The mouse was deeply anesthetized with sodium pentobarbital (100 mg/kg by intraperitoneal injection). When there was no response to tail and paw pinches, the mouse was transcardiacally perfused with cold (4–8 °C) dissection solution before decapitation. The brain was quickly removed and placed in ice-cold dissection solution for 1–2 min for further cooling, and then glued to a metal block with the dorsal (cortical) side facing down. Horizontal brain slices were cut on a vibratome (VT1200; Leica Biosystems, ON, Canada) and collected in ice-cold dissection solution. After vibratome sectioning, the slices were maintained in oxygenated ACSF for ≥ 45 min before further manipulation. The components of dissection solution were: 280 mM sucrose, 3.5 mM KCl, 0.5 mM CaCl_2_, 6 mM MgCl_2_, 10 mM HEPES, and 10 mM d-glucose (pH 7.35–7.40 titrated with NaOH).

### Recording apparatus

All recordings were performed in a custom submerged chamber with inner dimensions of 3.5 × 5 × 20 mm (depth × width × length) (Wu et al. 2002) [40, 41]. The hippocampal slice was held on a stainless- steel fine mesh (0.015-inch grid length) via a frame made of fine stainless-steel wires. The mesh was set 1.5 mm above the bottom of the chamber to allow perfusion of both sides of the slice with oxygenated (95% O_2_/5% CO_2_) sACSF or mACSF at a high rate (~ 15 ml per min) and a perfusate temperature of 35–36 °C. Care was taken to keep the slice at a minimal submerged level to achieve effective exchange with the oxygenated ACSF. A water bath underneath the recording chamber was set at 35 °C via an automatic temperature control unit, and humidified gas (95% O_2_/5% CO_2_) was passed over the perfusate to increase local oxygen tension in the recording chamber. To perfuse slices with warmed ACSF, aerated (95% O_2_/5% CO_2_) ACSF was warmed to 35–36 °C using a large warm bath with automatic temperature control and added to a perfusion bottle that was surrounded by an automatic heating blanket to maintain the temperature at 35–36 °C. Under these conditions, the perfusate temperature as measured by a fine temperature probe near the perfused slice was close to (≤ 0.5 °C) the ACSF temperature in the perfusion bottle [40, 41]. Previous work from our laboratory and others has shown that rapid perfusion of both sides of rodent brain slices is important for maintaining spontaneous population activity under submerged recording conditions [14, 40, 41], Such recording settings, by providing sufficient oxygenation and glucose delivery to slice tissues, are conceivably critical to sustain epileptiform potentials particularly ictal-like discharges with high energy demand [31].

### Recordings and stimulating electrodes

Electrodes for extracellular recording were pulled from thin-walled glass tubes (World Precision Instruments, Sarasota, FL, USA) using a vertical puller (PS-88; Narishige International USA, Amityville, NY, USA). The electrodes were filled with a solution of 150 mM NaCl and 2 mM HEPES (pH 7.4; resistance of 1–2 MΩ). A 2-channel amplifier (Multiclamp 700A; Molecular Devices, Sunnyvale, CA, USA) was used to record field potentials. The amplifier was set with an input frequency band of 0–3000 Hz and amplification gain of 50–100. The output signals of the amplifier were digitized at 5000–10,000 Hz (Digidata 1400 or 1500; Molecular Devices). Clampex (pClamp) v10 software (Molecular Devices) was used for data acquisition and storage.

A twisted wire bipolar electrode composed of polyamide-insulated stainless-steel wire (110 μm outer diameter; Plastics One, Ranoake, VA, USA) was used for afferent stimulation. The stimulating electrode was positioned in the CA3 stratum oriens aera. Constant current pulses (0.5-ms duration at a near-maximal intensity of 150 μA) were generated with a Grass stimulator (S88; Grass Medical Instruments, Warwick, RI, USA) and delivered via a photoelectric isolation unit. Synaptic field potentials were evoked every 20 s. HFS was applied at 100 Hz for 1 s repeated 10 times at a 1-s interval [19].

### Data analysis

Evoked and spontaneous field potentials were analyzed using Clampfit software. Peak amplitudes of evoked filed potentials were measured from an average of 4–5 consecutive responses. Interictal-like spikes were automatically detected using the event detection function (threshold search method) of Clampfit. Events with peak amplitudes ≥ 6 times the standard deviation of the baseline signal were detected, and visually inspected, and false events were rejected. If necessary, original data were treated with a Bessel high-pass filter (1–2 Hz, 8-pole) before automatic event detection. Origin v2020 software (Origin Lab, Northampton, MA, USA) was used for cross-correlation analysis of discharge signals after treatment with a high-pass filter (2 Hz).

Only slices with evoked CA3 responses ≥ 0.5 mV in initial recordings were retained for further analysis. As evoked field potentials represent local circuit activity and are sensitive to experimental perturbations, we used this selection approach for both control and mACSF-pretreated slices to mitigate the potential influence of dissection-related tissue damage. We randomly selected slices that were successfully prepared from each mouse for recording to control for the potential effect of time-dependent slice deterioration on the generation of epileptiform field potentials.

### Chemicals and pharmacologic agents

All chemicals for preparing ACSF and other solutions as well as the glutamate receptor antagonists CNQX, AP5, and MK801 were obtained from Sigma-Aldrich (Oakville, ON, Canada). Sodium phenytoin in a clinically injectable form was obtained from Sandoz (Boucherville, QC, Canada). All solutions were made of deionized distilled water (specific resistance ≥ 18 MΩ, Milli-Q CLX 7000; Sigma-Aldrich). CNQX and MK801 were dissolved in dimethylsulfoxide to obtain stock solutions of 10 or 20 mM. AP5 was dissolved in water to obtain a stock solution of 10 mM. All stock solutions were stored at − 20 °C until use. Phenytoin, CNQX, AP5, and MK801 were diluted in mACSF to the desired concentration and applied by bath perfusion.

### Statistical analysis

Statistical analyses were performed using SigmaPlot software (Systat, San Jose, CA, USA) or Origin software. Data are presented as mean ± standard error of the mean. For normally distributed data, intergroup differences were assessed with the student’s t test or by one-way analysis of variance (ANOVA) followed by a Bonferroni post hoc test. For non-normally distributed data, the Mann–Whitney U test or nonparametric ANOVA on ranks (Kruskal–Wallis test) followed by a post hoc test was used for intergroup comparisons. The chi-squared test or Fisher’s exact test was used for rate comparisons. Statistical significance was set at p < 0.05.

## Supplementary Information

**Additional file 1: Fig. S1.** Hippocampal interictal spikes induced by moderately modified (m)ACSF. Data collected from a thin slice of a middle-aged mouse. The slice was pretreated with mACSF and perfused with mACSF during recordings. **A** Extracellular field potentials recorded simultaneously from CA3 and CA1 areas and illustrated after treatment with a band-pass filter (2–500 Hz). **B** Arrowed events expanded in a wide frequency band (0–1000 Hz). Superimposed gray traces demonstrated 10 consecutive interictal spikes (denoted by a gray box in A). Averages from the 10 events presented by dark traces. **C** Traces in B illustrated after treatment with a ban-pass filter (50–500 Hz). Note CA3 oscillatory activities of roughly 230–250 Hz in the 2nd and last panel.

**Additional file 2: Fig. S2.** Hippocampal epileptiform activities induced by moderately modified (m)ACSF. Data collected from two thin hippocampal slices of two young mice. Extracellular filed potentials recorded simultaneously from CA3 and CA1 areas. During recording, slices perfused with standard ACSF (sACSF) for 20 min and then with mACSF. The time of sACSF or mACSF perfusion denoted by a dashed or solid line above traces, respectively. **A** Original signals illustrated after treatment with a band-pass filter (2–500 Hz). Note no evident epileptiform activity following sACSF perfusion and ictal discharge appeared ~ 8 min following mACSF perfusion. **B** Arrowed CA3 and CA1 ictal discharges expanded in a wide frequency band (0–1,000 Hz). **C** Traces similarly illustrated as in A. Note appearance of interictal spikes ~ 9 min following mACSF perfusion. **D**, arrowed events illustrated in in a wide frequency band (0–1000 Hz) and showed CA3 unit spikes (left) and CA3 and CA1 interictal spikes (right).

**Additional file 3: Fig. S3.** Piriform discharges induced by moderately modified (m)ACSF. Traces collected from two thin slices of young mice via extracellular recordings in piriform areas. **A** Field potentials recorded following mACSF perfusion and illustrated in a wide frequency band (0–1000 Hz). Note appearance of spontaneous ictal discharges with incremental amplitudes. An arrowed event expanded to show complex discharge waveform. **B** Filed potential collected before and following high frequency stimulation (HFS, filled arrow) and similarly illustrated as above. Note appearance of ictal discharges ~ 4 min after HFS and displayed incremental amplitudes.

## Data Availability

The datasets used and/or analyzed during the current study are available from the corresponding author on reasonable request.

## Abbreviations

ACSF: Artificial cerebrospinal fluid
mACSF: Moderately modified ACSF
ANOVA: One-way analysis of variance
HFS: High frequency stimulation
CNQX: 6-Cyano-7-nitroquinoxaline-2,3-dione
AP5: 2-Amino-5-phosphonopentanoate
SE: Standard error of the mean
