# Supplementary figures and images for: Epileptiform activity in mouse hippocampal slices induced by moderate changes in extracellular Mg2+, Ca2+, and K+
Source: BMC Neurosci. 2021 Jul 23;22:46. doi: 10.1186/s12868-021-00650-3 (PMC8305515; doi:10.1186/s12868-021-00650-3)

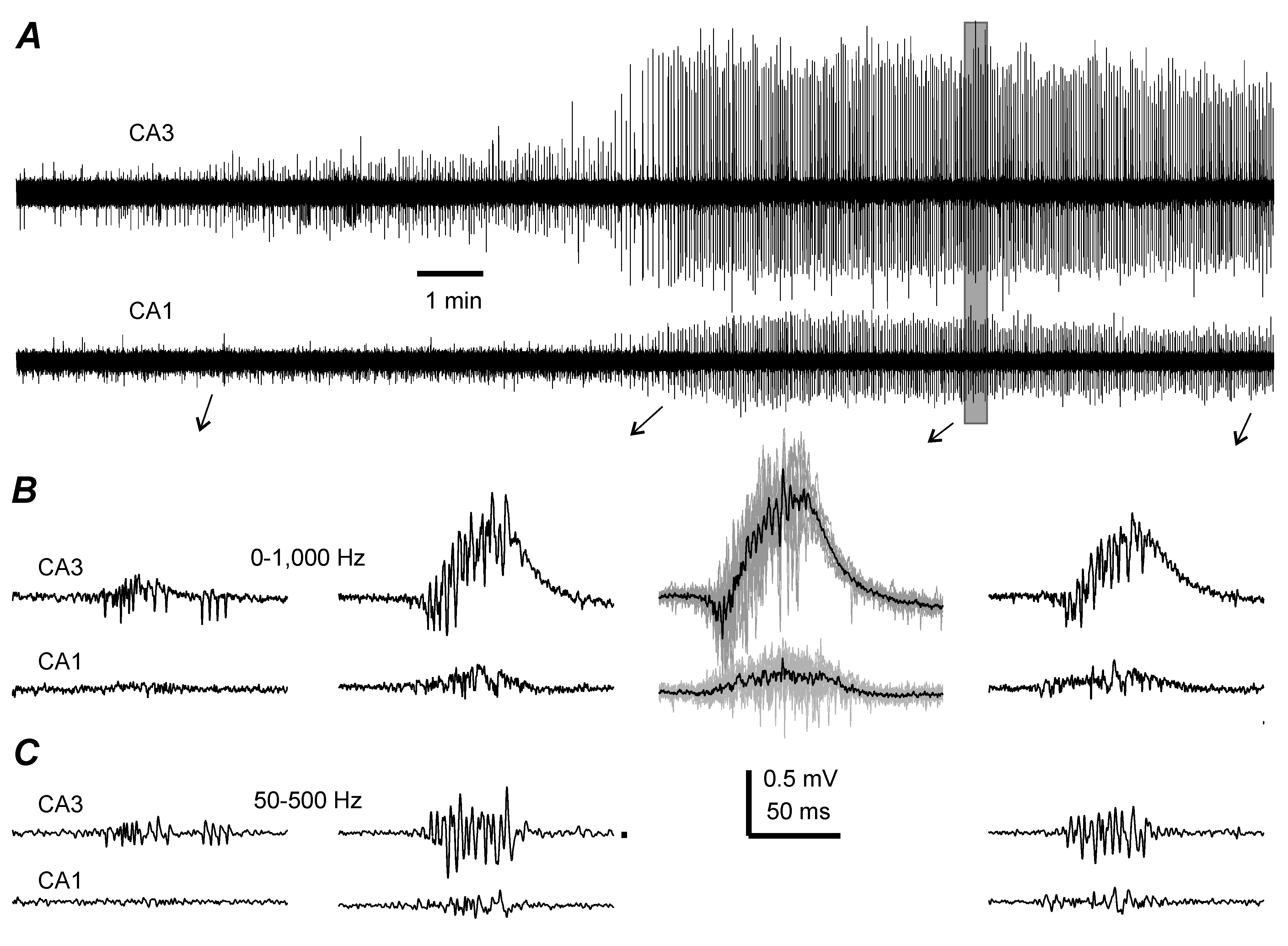

Supplement: Supplementary file 1 — Additional file 1: Fig. S1. Hippocampal interictal spikes induced by moderately modified (m)ACSF. Data collected from a thin slice of a middle-aged mouse. The slice was pretreated with mACSF and perfused with mACSF during recordings. A Extracellular field potentials recorded simultaneously from CA3 and CA1 areas and illustrated after treatment with a band-pass filter (2–500 Hz). B Arrowed events expanded in a wide frequency band (0–1000 Hz). Superimposed gray traces demonstrated 10 consecutive interictal spikes (denoted by a gray box in A). Averages from the 10 events presented by dark traces. C Traces in B illustrated after treatment with a ban-pass filter (50–500 Hz). Note CA3 oscillatory activities of roughly 230–250 Hz in the 2nd and last panel. [file 12868_2021_650_MOESM1_ESM.tif]

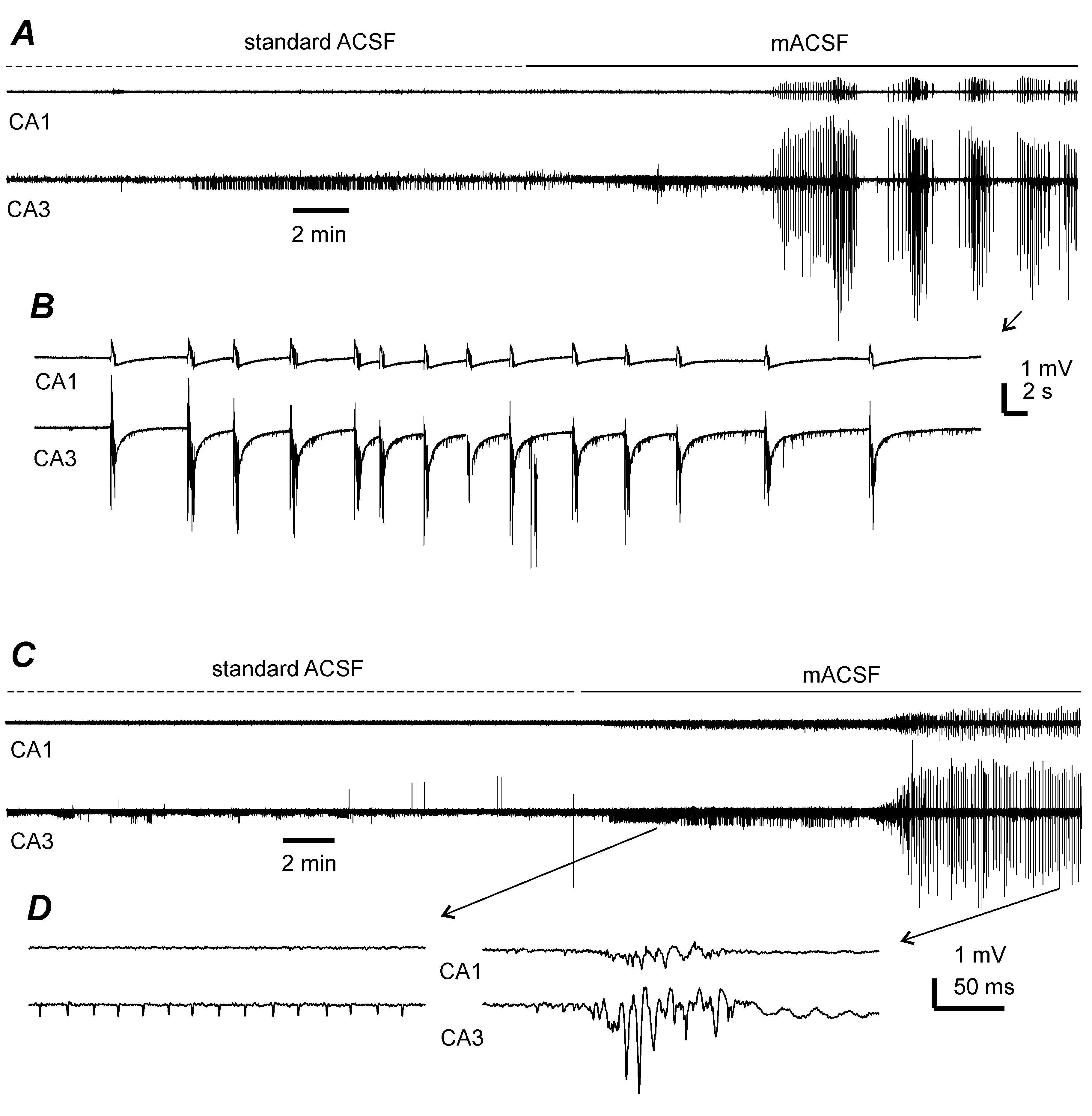

Supplement: Supplementary file 2 — Additional file 2: Fig. S2. Hippocampal epileptiform activities induced by moderately modified (m)ACSF. Data collected from two thin hippocampal slices of two young mice. Extracellular filed potentials recorded simultaneously from CA3 and CA1 areas. During recording, slices perfused with standard ACSF (sACSF) for 20 min and then with mACSF. The time of sACSF or mACSF perfusion denoted by a dashed or solid line above traces, respectively. A Original signals illustrated after treatment with a band-pass filter (2–500 Hz). Note no evident epileptiform activity following sACSF perfusion and ictal discharge appeared ~ 8 min following mACSF perfusion. B Arrowed CA3 and CA1 ictal discharges expanded in a wide frequency band (0–1,000 Hz). C Traces similarly illustrated as in A. Note appearance of interictal spikes ~ 9 min following mACSF perfusion. D, arrowed events illustrated in in a wide frequency band (0–1000 Hz) and showed CA3 unit spikes (left) and CA3 and CA1 interictal spikes (right). [file 12868_2021_650_MOESM2_ESM.tif]

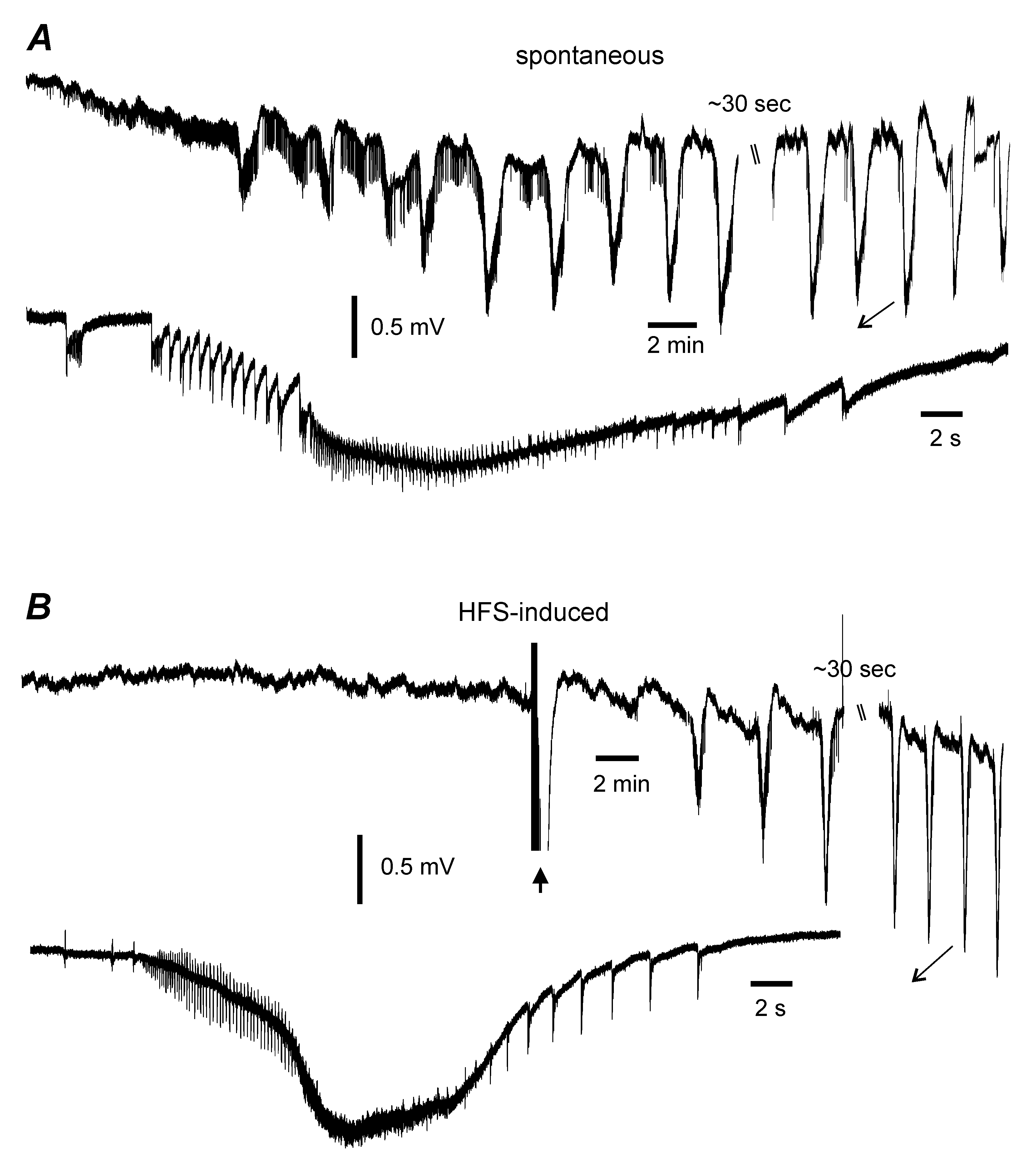

Supplement: Supplementary file 3 — Additional file 3: Fig. S3. Piriform discharges induced by moderately modified (m)ACSF. Traces collected from two thin slices of young mice via extracellular recordings in piriform areas. A Field potentials recorded following mACSF perfusion and illustrated in a wide frequency band (0–1000 Hz). Note appearance of spontaneous ictal discharges with incremental amplitudes. An arrowed event expanded to show complex discharge waveform. B Filed potential collected before and following high frequency stimulation (HFS, filled arrow) and similarly illustrated as above. Note appearance of ictal discharges ~ 4 min after HFS and displayed incremental amplitudes. [file 12868_2021_650_MOESM3_ESM.tif]
